# Blind Speech Watermarking Method with Frame Self-Synchronization Based on Spread-Spectrum Using Linear Prediction Residue

**DOI:** 10.3390/e24050677

**Published:** 2022-05-11

**Authors:** Takuto Isoyama, Shunsuke Kidani, Masashi Unoki

**Affiliations:** School of Information Science, Japan Advanced Institute of Science and Technology, Ishikawa 923-1292, Japan; kidani@jaist.ac.jp (S.K.); unoki@jaist.ac.jp (M.U.)

**Keywords:** speech watermarking, linear prediction, spread spectrum, liner prediction residue, blind detection, frame synchronization

## Abstract

State-of-the-art speech watermarking techniques enable speech signals to be authenticated and protected against any malicious attack to ensure secure speech communication. In general, reliable speech watermarking methods must satisfy four requirements: inaudibility, robustness, blind-detectability, and confidentiality. We previously proposed a method of non-blind speech watermarking based on direct spread spectrum (DSS) using a linear prediction (LP) scheme to solve the first two issues (inaudibility and robustness) due to distortion by spread spectrum. This method not only effectively embeds watermarks with small distortion but also has the same robustness as the DSS method. There are, however, two remaining issues with blind-detectability and confidentiality. In this work, we attempt to resolve these issues by developing an approach called the LP-DSS scheme, which takes two forms of data embedding for blind detection and frame synchronization. We incorporate blind detection with frame synchronization into the scheme to satisfy blind-detectability and incorporate two forms of data embedding process, front-side and back-side embedding for blind detection and frame synchronization, to satisfy confidentiality. We evaluated these improved processes by carrying out four objective tests (PESQ, LSD, Bit-error-rate, and accuracy of frame synchronization) to determine whether inaudibility and blind-detectability could be satisfied. We also evaluated all combinations with the two forms of data embedding for blind detection with frame synchronization by carrying out BER tests to determine whether confidentiality could be satisfied. Finally, we comparatively evaluated the proposed method by carrying out ten robustness tests against various processing and attacks. Our findings showed that an inaudible, robust, blindly detectable, and confidential speech watermarking method based on the proposed LP-DSS scheme could be achieved.

## 1. Introduction

The rapid development of information and communication technology (ICT) has led to a positive effect on societies and communities in many ways, along with a dramatic increase in multimedia information usage via the Internet. In addition, new innovations have appeared alongside the utilization of big data collected from cyber-physical systems and worldwide networks. Although it can enrich our daily lives, multimedia big data, which includes personal data that users wish to keep private, is at high risk of illegal distribution and misuse through the proliferation of non-authentic media and the leakage of private information.

For example, speech communication technology has been implemented via speech communication channels such as the Voice over Internet Protocol (VoIP) and the Public Switched Telephone Network (PSTN), and since these channels are considerably vulnerable to attacks, problems such as speech tampering, spoofing, and issues regarding the digital forensics of speech data using voice conversion and text-to-speech techniques have occurred [1,2]. Therefore, it is necessary to achieve secure protection and implement preventative countermeasures in speech communication technology.

The speech watermarking technique has attracted attention as a solution to achieve these countermeasures against speech tampering and spoofing [3,4]. This technique aims to protect digital speech content by embedding an inaudible security code into a speech signal and by detecting the embedded security code from the watermarked speech signal. In general, speech watermarking methods must meet four requirements to provide a useful and reliable form of audio/speech watermarking [2,5]: (1) inaudibility (inaudible to humans with no sound distortion caused by the embedded data), (2) robustness (not affected when subjected to techniques such as data compression and malicious attacks), (3) blind-detectability (high possibility of detecting the embedded data without using the original or reference signal), and (4) confidentiality (secure and undetectable concealment of embedded data).

Conventional audio watermarking techniques can also be used as speech watermarking [6]. Typical examples include the least-significant bit (LSB) replacement method [3] and the direct spread spectrum (DSS) method [3,7]. The LSB method has an advantage concerning inaudibility because it has less effect on the magnitude [3] but a disadvantage regarding fragility against any modifications. In contrast, the DSS method has an advantage regarding robustness against various modifications [3,7] but a disadvantage regarding inaudibility due to sound distortion by spectrum spreading. In short, the LSB method satisfies requirement (1) but not (2), and the DSS method satisfies requirement (2) but not (1).

Other state-of-the-art audio watermarking methods include singular value decomposition (SVD) with dither modulation quantization, which is a type of quantization index modulation (QIM) [8,9], and various phase modulation techniques [5,10]. Although they have strong points in terms of one or two requirements (e.g., inaudibility and robustness or blind-detectability), they cannot satisfy all four requirements simultaneously due to fragility against speech codecs and sensitivity to frame desynchronization attacks. This suggests that a speech watermarking method using typical audio watermarking techniques must be reconsidered to satisfy the robustness against speech codecs and to ensure blind detection with frame synchronization.

There are some specific speech watermarking methods that depend on speech codecs [2]. These methods are individually designed by adaptively embedding and detecting a security code into/from parameters (linear predictive parameters, codebook parameters, vector quantization, etc.) in the speech codec scheme for G.711, G.721, G.728, G.729, and GSM, although the main approach is taken into code excited linear prediction (CELP) and mixed excitation linear prediction (MELP). These methods can be used for covert communication based on the VoIP system and the design of real-time communication over PSTN [2]. All of these methods have some kind of trade-off between sound quality, capacity, and robustness. However, as there have been no comparative evaluations of these methods under various conditions, it is unclear whether or not they can satisfy all four requirements simultaneously, although it is safe to say that they are not robust against various speech codecs because they depend on specific speech codecs.

A few sophisticated speech watermarking methods based on the source-filter model have been proposed for application to speech tampering detection [11,12]. The source-filter model assumes the glottal pulse is the sound source and the vocal tract is a filter (spectral envelope) in a speech synthesis system. Linear prediction (LP) synthesis can be utilized as the source-filter model. In the LP synthesis method, the speech into sound source as LP residue and the vocal tract information as LP coefficients are used to synthesize speech signals. This inspired us to investigate whether a speech watermarking-based LP scheme could be implemented by separately applying watermarking to LP residues as the source and LP coefficients as the vocal tract filter without affecting each other.

Speech watermarking methods based on formant tuning (FT) [13] and on the McAdams coefficient (MC) [14] have been successfully proposed for LP schemes to embed the security code into the spectral envelope of a speech signal, i.e., LP coefficients. The FT method achieves inaudible watermarking by controlling line spectral frequency (LSF) features converted from the LP coefficients to embed the security code into formant tuning. The MC-based method also achieves inaudible robust watermarking by controlling McAdams coefficients to embed the security code into frequency spectral scaling, i.e., scale-shifting for spectral envelope shapes related to the LFSs. These methods satisfy robustness because the LSF features are robust against various speech codecs. Although both methods can satisfy blind-detectability, they may be sensitive to frame desynchronization issues. Confidentiality in these methods can be satisfied by specifying a non-public condition in the watermarking algorithms.

Another possibility for considering speech watermarking based on the LP scheme is to embed the security code into LP residue. We previously proposed a DSS method using LP residue (LP-DSS scheme) to combine the robustness of the DSS method with the inaudibility of the LP-based method [15], which was inspired by the similarity between the statistical properties of the pseudo random noise (PN) signal used in DSS and the LP residue. While this method was more inaudible than the DSS method and kept the same robustness, it did not satisfy the last two requirements, blind-detectability and confidentiality, because it is still non-blind speech watermarking.

This paper aims to develop a state-of-the-art speech watermarking technique that satisfies all four requirements. To this end, we propose a blind speech watermarking method based on the LP-DSS scheme that incorporates its blind detection and frame synchronization and adds two embedding processes to solve the blind-detectability and confidentiality issues.

This paper is organized as follows. Section 2 describes the LP-DSS scheme and then addresses the remaining issues. Section 3 describes the two forms of data embedding for blind detection and frame synchronization to solve the remaining issues. Section 4 studies validation of the proposed method with regard to frame synchronization and blind detection, and then provides how to set embedding strength in the proposed method. Section 5 comparatively evaluates the proposed method by comparison with other related three methods with regard to the four requirements for speech watermarking. Section 6 concludes this paper.

## 2. Non-Blind LP-DSS Watermarking Scheme

### 2.1. Concept

The DSS method is implemented by spectrally spreading the security code m(n) with the PN signal c(n) and embedding it in the host signal x(n), as

(1) $$\begin{array}{ccc} y(n) & = & x(n)+am(n)c(n), \end{array}$$

where *a* is the amplitude term to adjust the embedding strength and *n* is the samples. The PN signal c(n) has the properties E{c(n)}=0 and $E\{c^{2}\left(n\right)\}=1$. E{·} is the expectation operator for “·”.

Linear predictive coding (LPC) is one of the most basic speech coding methods and is based on LP. It uses the LP coefficients and the LP residue r(n) obtained by LP analysis, as

(2) $$\begin{array}{c} \hat{x}\left(n\right)=\sum_{i=1}^{P}\alpha_{i}x\left(n-i\right), \end{array}$$

(3) $$\begin{array}{c} r\left(n\right)=x\left(n\right)-\hat{x}\left(n\right), \end{array}$$

where $\hat{x}\left(n\right)$ is the predicted speech, $\alpha_{i}$ is the LP coefficients, and *P* is the LP order. The LP residue r(n) has the properties E{r(n)}=0 and $E\{r^{2}\left(n\right)\}=1$, which are the same as those of the PN signal used in the DSS method. On the basis of these properties, the DSS method can also be implemented using the LP residue instead of the PN signal. For this reason, we refer to the proposed method based on this concept as the LP-DSS scheme.

### 2.2. Data Embedding and Detection

**Figure 1:**
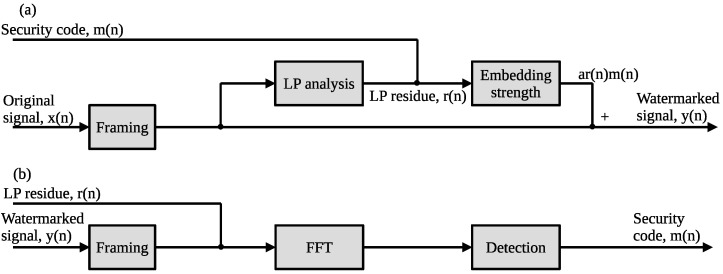
Block diagram of LP-DSS method: (**a**) embedding and (**b**) detection.

Figure 1 shows a block diagram of the speech watermarking method based on the LP-DSS scheme, where (a,b) show the embedding process and the detection process for the security code, respectively. In Figure 1a, x(n) is divided into *K* frames by framing it with a rectangular window function, where *K* is the number of frames obtained by dividing the signal length by the window length used in the LP analysis. In each frame, the watermark m(n)r(n) are obtained by multiplying the security codes m(n) by the LP residue r(n), which is the spreading signal by m(n). The watermarked signal y(n) is obtained by embedding m(n)r(n) into the host signal x(n) in each frame, as

(4) $$\begin{array}{ccc} y(n) & = & x(n)+am(n)r(n), \end{array}$$

(5) $$\begin{array}{ccc} a & = & 10^{L_{\mathrm{all}}/20}, \end{array}$$

(6) $$\begin{array}{ccc} L_{\mathrm{all}} & = & L_{\mathrm{PHS}}-L_{\mathrm{PWS}}+L_{\mathrm{SSL}}, \end{array}$$

where *a* is the amplitude term to adjust the embedding strength, $L_{\mathrm{PHS}}$ is the power level of the host signal, $L_{\mathrm{PWS}}$ is the power level of the signal with the embedded security code, and $L_{\mathrm{SSL}}$ is the embedding-strength level in dB. The security code m(n) is defined as

(7) $$m\left(n\right)=\left\{\begin{array}{c} 0,\;\;E\{y(n)r(n)\}\leq 0\\ 1,\;\;E\{y(n)r(n)\}>0 \end{array}\right.,$$

where x(n), y(n), and r(n) are assumed to be ergodic. The LP residue has the statistical properties E{r(n)}=0 and $E\{r^{2}\left(n\right)\}=1$. The security code m(n) is detected by multiplying the watermark signal y(n) by the LP residue r(n) again to obtain the expected value E{y(n)r(n)} using the Fourier transform. The security code m(n) is calculated as

(8) $$\begin{array}{ccc} E\{y(n)r(n)\} & = & E\{[x(n)+am(n)r(n)]r(n)\}\\ & = & E\left\{x\left(n\right)r\left(n\right)\right\}+E\left\{am\left(n\right)r^{2}\left(n\right)\right\} \end{array}$$

(9) $$\begin{array}{ccc} & = & am(n), \end{array}$$

where E{am(n)} is am(n), and x(n) and r(n) are mutually orthogonal, so the first term in Equation (8) is 0. The second term is $E\{am\left(n\right)r^{2}\left(n\right)\}=am\left(n\right)$.

We investigated whether the LP-DSS method can satisfy the two requirements of inaudibility and robustness by comparing it with the DSS method. The non-blind LP-DSS method was evaluated by carrying out three objective tests (perceptual validation of speech quality (PESQ), log-spectrum distortion (LSD), and bit-error-rate (BER) for various modifications to the watermarked signal) for inaudibility and robustness on 12 utterances in the Advanced Telecommunications Research Institute International (ATR) speech dataset (B set) [16]. The LP order is 12, the embedding bit rates are 4, 8, 16, 32, and 64 bps, and the embedding strength level $L_{\mathrm{SSL}}$ is 10 dB. The results of these three tests showed that the LP-DSS method could embed watermarks with low sound quality between 4 and 16 bps and that it had the same robustness as the DSS method.

### 2.3. Remaining Issues

The non-blind LP-DSS method has two remaining issues: blind-detectability and confidentiality.

Blind-detectability: A high possibility of detecting the embedded data without using the original or reference signal must be satisfied. In voice communication channels such as VoIP and PSTN, the watermarked signal and the host signal cannot be sent simultaneously. Therefore, the non-blind LP-DSS method cannot be used for VoIP and PSTN. For the LP-DSS method to achieve blind detection, the LP residue used for embedding must be robust and accurate. Even if the LP residue is obtained from the watermarked signal again by LP analysis, the accuracy depends on the embedding-strength level. If the embedding-strength level is −20 dB, y(n) is nearly equal to x(n), and the accurate LP residue can be obtained from y(n). Needless to say, it is difficult to detect the security code robustly and accurately when the embedding strength is low. LP analysis is not robust to noise, so the LP residue cannot be obtained accurately when the embedding strength is very high. In addition, this method is processed on a frame-by-frame basis, which means the security code cannot be detected correctly if the position of the embedded security code is different from the position of the detected security code. To solve this problem, we need to synchronize the frames accurately and blindly to detect the security code. Therefore, it is necessary to inform the detection side of the synchronized position.

Confidentiality: Secure and undetectable concealment of embedded data must be satisfied. The non-blind LP-DSS method uses LP residue instead of the PN signal used in the DSS method, which means it can satisfy the confidentiality of the watermarking algorithm under non-public conditions. However, security codes are easily detected in public conditions because LP residues can possibly be obtained from the watermarked signals. The DSS method uses a key to create the PN signal to enhance confidentiality in public environments, but this is limited to non-blind environments. Therefore, the non-blind LP-DSS method is less confidential under public conditions than the DSS method.

We investigated ways of addressing the blind-detectability and confidentiality to further develop the LP-DSS scheme.

## 3. Proposed Method

We propose blind detection and frame synchronization processes to satisfy the blind-detectability requirement. We also proposed two forms of data embedding for the blind detection to satisfy the confidentiality requirement.

We assume that the LP residues of three adjacent frames are highly correlated. This suggests that the LP residue of an even-numbered frame can be used to embed security codes in odd-numbered frames, and then the LP residue of the even-numbered frame can be used to accurately detect security codes from the watermarked signal of the odd frames. We also speculate that the opposite embedding/detection form can be used by interchanging the even-numbered/odd-numbered frames. Given that the LP residues used for embedding and detection are the same, the frame synchronization process can also be taken into account by maximizing the cross-correlation between the watermarked signal and the LP residue as a norm.

### 3.1. Concept of Data Embedding for Blind Detection and Frame Synchronization

The LP scheme presupposes that the time sequence signal is a stationary process. Within a short duration, speech signals are stationary, so they can be accurately predicted using the LP scheme. Consequently, the host signals of adjacent frames have equal statistical properties during frame processing, and the host signals are highly correlated across adjacent frames. The LP residues obtained from the host signal are also expected to be highly correlated across adjacent frames.

LP residue $r_{k}\left(n\right)$ in the (*k*)th frame is assumed to be equal to LP residue in adjacent frames in the (k−1)th frame or (k+1)th frame. On the basis of this assumption, the LP residue in the adjacent frame in the (k−1)th frame or (k+1)th frame can be used to embed a security code in the host signal $x_{k}\left(n\right)$ in the (*k*)th frame. The security code can also be detected by using the LP residue in adjacent frames in the (k−1)th frame or (k+1)th frame. Furthermore, if we consider the embedding of security code in the forms of front and back frames, when the LP residue $r_{k}\left(n\right)$ in the (*k*)th frame for embedding and the LP residue $r_{k}^{\prime }\left(n\right)$ in the (*k*)th frame for detection are the same ($r_{k}\left(n\right)=r_{k}^{\prime }\left(n\right)$), the expectation is calculated as $E\{r_{k}^{2}\left(n\right)\}=1$.

On the other hand, when the different LP residue ($r_{k}\left(n\right)\neq r_{k}^{\prime }\left(n\right)$) in the (*k*)th frame is used for embedding and detecting secure code into a speech signal, the expected value is $E\{r_{k}\left(n\right)r_{k}^{\prime }\left(n\right)\}<<1$. The expected value $E\{r_{k}\left(n\right)r_{k}^{\prime }\left(n\right)\}$ in the case of the frame being correctly synchronized is thus higher than in the case of the frame not being correctly synchronized.

**Figure 2:**
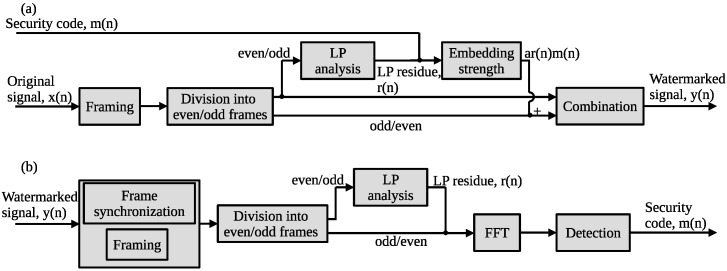
Block diagram of proposed method: (**a**) embedding process and (**b**) detection process with frame synchronization.

Figure 2 shows the block diagram of the proposed method, where (a,b) show the embedding and detection processes for the security code, respectively. The difference between the non-blind LP-DSS method and the blind LP-DSS method is that the host signal is classified into even-numbered and odd-numbered frames, and the LP residue from the host signal of the even-numbered or odd-numbered frame is used to embed the security code in the host signal of other frames. This process enables the LP residue to be processed from the watermarked signal of the unprocessed frame and the security code to be detected from the watermarked signal of the other frame. In addition, even if the frame synchronized position is shifted during the security code detection, the proposed method can correctly detect the security code by synchronizing the frames. The data embedding, the frame synchronization, and the blind detection processes are explained in Section 3.2–Section 3.4, respectively.

### 3.2. Data Embedding Process

**Figure 3:**
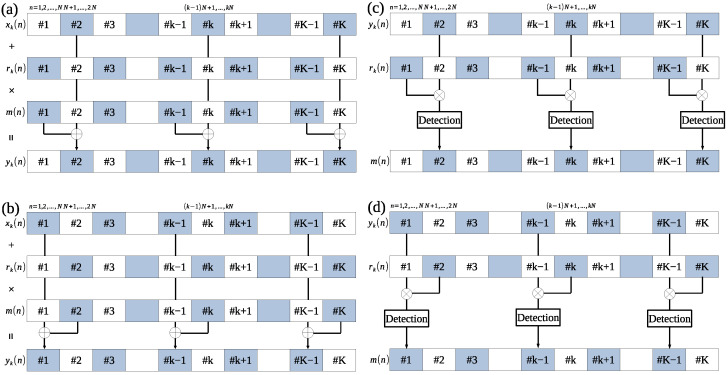
Block diagram of proposed blind-detection method. Security code embedding in (**a**) front-side and (**b**) back-side forms. Security code detection in (**c**) front-side and (**d**) back-side form.

As a basic assumption, the frame length is known. We consider two forms of embedding process in the frame processing: front-side and back-side. Figure 3a,b show the embedding process of the security code in the front-side and back-side forms, respectively.

The host signal is divided into *K* frames by a short-term analysis using a fixed frame length. The even-numbered frame was set to *k* and the host signal of this frame was set to $x_{k}\left(n\right)$ in the embedding process of the front-side form. In this case, the LP residue $r_{k-1}\left(n\right)$ in the (k−1)th frame is obtained by LP analysis from the host signal $x_{k-1}\left(n\right)$ in the odd-numbered frame. The security code m(n) is spread-spectrum modulated using the LP residue $r_{k-1}\left(n\right)$ in the (k−1)th frame and embedded in the host signal $x_{k}\left(n\right)$ of the even-numbered frame. The watermarked signal is $y_{k}\left(n\right)$. The watermarked signal $y_{k-1}\left(n\right)$ in the even-numbered frame is the same as the host signal $x_{k-1}\left(n\right)$ in the even-numbered frame. To embed the security code robustly, the embedding strength *a* is controlled by the embedding-strength level $L_{\mathrm{SSL}}$ (as in the LP-DSS method) using Equation (6).

Similarly, the embedding process (back-side form) can be done by switching the even/odd-numbered frames for LP residue calculation and embedding, as shown in Figure 3b.

### 3.3. Frame Synchronization Process

**Figure 4:**
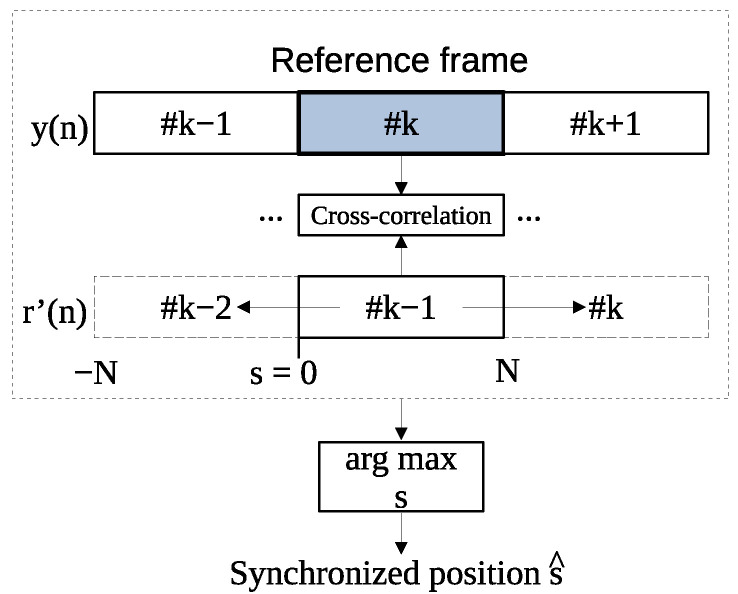
Proposed frame-synchronization process when the number of reference frames is one. When the number of reference frames is two or more, this process obtained the frame synchronization position by arithmetic averaging of the cross-correlation results.

Figure 4 shows the proposed frame synchronization process. The watermarked signal is segmented into *K* frames. LP residue $r_{k-1}\left(n\right)$ in the (k−1)th frame is calculated from the watermarked signal $y_{k-1}\left(n\right)$ in the (k−1)th frame. The expected value is obtained by the following equation to multiply the obtained LP residue $r_{k-1}^{\prime }\left(n\right)$ in the (k−1)th frame by the watermarked signal $y_{k}\left(n\right)$ in the (*k*)th frame.

(10) $$\begin{array}{ccc} F_{k}\left(s\right) & = & E\{y_{k}\left(n-s\right)r_{k-1}^{\prime }\left(n-s\right)\}. \end{array}$$

Substituting $y_{k}\left(n-s\right)=x_{k}\left(n-s\right)+am\left(n-s\right)r_{k-1}\left(n-s\right)$ into Equation (10) yields

(11) $$\begin{array}{ccc} F_{k}\left(s\right) & = & E\left\{x_{k}\left(n-s\right)r_{k-1}^{\prime }\left(n\right)\right\}+E\left\{am\left(n-s\right)r_{k-1}\left(n-s\right)r_{k-1}^{\prime }\left(n\right)\right\}, \end{array}$$

where $x_{k}\left(n-s\right)$, $y_{k}\left(n-s\right)$, and $r_{k-1}\left(n-s\right)$ are assumed to be ergodic. The LP residue has the statistical properties $E\{r_{k-1}\left(n-s\right)\}=0$ and $E\{r_{k-1}\left(n-s\right)\}=1$. We then multiply the watermarked signal $y_{k}\left(n-s\right)$ by the LP residue $r_{k}^{\prime }\left(n-s\right)$ and obtain the expected value $E\{y_{k}\left(n-s\right)r_{k-1}^{\prime }\left(n-s\right)\}$ by Fourier transform as follows:

(12) $$\begin{array}{ccc} F_{k}\left(s\right) & = & E\{am\left(n-s\right)r_{k-1}\left(n-s\right)r_{k-1}^{\prime }\left(n-s\right)\}, \end{array}$$

where *s* is the sample shift: s=−N,−N+1,···,−1,0,1,···,N−1,N. The subscript *k* is the reference frame that is defined as frames of watermark signal y(n): k=2,4,6,···,K−2,K. The $r_{k-1}\left(n-s\right)$ is the LP residue in the (k−1)th frame used to embed the security code. By finding the expectation value as in Equation (9), only the second term in Equation (11) remains. Then, the watermarked signal $y_{k}\left(n-s\right)$ in the (*k*)th frame is shifted by one sample and the expected value is calculated in the same way. This is done until s=−N∼N samples. The expected value is processed as an absolute value, and the arithmetic mean is calculated using the correlation values for the number of reference frames, as

(13) $$\begin{array}{ccc} \bar{F}\left(s\right) & = & \frac{2}{K}\sum_{k=2,k\in \mathrm{even}}^{K}\left|F_{k}\left(s\right)\right|. \end{array}$$

The synchronized position $\hat{s}$ is determined from the maximum value of $\bar{F}$ obtained from the arithmetic mean, as

(14) $$\begin{array}{c} \hat{s}=\underset{s}{\arg\max}\bar{F}\left(s\right). \end{array}$$

### 3.4. Blind Detection Process

We consider two forms of detection process in the frame processing: front-side and back-side. Figure 3c,d show the detection process of the security code in the front-side and back-side forms, respectively.

The security code is detected from the watermarked signal $y_{k}\left(n\right)$ in the (*k*)th frame. The watermarked signal $y_{k-1}\left(n\right)$ in the (k−1)th frame is the same as the host signal $x_{k-1}\left(n\right)$ in the (k−1)th frame in the detection process of the front-side form. The LP residue $r_{k-1}\left(n\right)$ in the (k−1)th frame is calculated from the watermarked signal $y_{k-1}\left(n\right)$ in the (k−1)th frame by using LP analysis. Then, the security code m(n) can be obtained accurately by multiplying LP residue $r_{k-1}\left(n\right)$ in the (k−1)th frame by $y_{k}\left(n\right)$ in the (*k*)th frame using the Equation (7).

Similarly, the detection process can be done backward (back-side form) by switching the even/odd-numbered frames for the LP residue calculation and detection, as shown in Figure 3d.

## 4. Validation of Proposed Method

In this section, we investigate whether the frame synchronization and blind detection processes are functioning as designed and determine the appropriate level for the embedding strength. The correct functioning of the frame synchronization process is evaluated by carrying out a frame synchronization test. The correct functioning of the blind detection process is evaluated by carrying out bit detection tests. The appropriate embedding-strength level is determined by examining the trade-off between PESQ, LSD, and BER.

### 4.1. Database and Conditions

The 12 utterances in the ATR database (B set) [16] were used to evaluate the frame synchronization process and blind detection process. The original speech signal had a 16-kHz sampling frequency, 16 bit quantization, 8.5 s duration, and one channel (mono).

The LP order *P* is 12 because the sampling frequency is 16 kHz. The frame length of the proposed method is 20 ms, the same as the frame length of the LP analysis. The bit rates (bps) in these validations were 4, 8, and 16 bps. Twelve random bit strings were used for each bit rate. The embedding-strength level for embedding the security code was set to −20∼0 dB in 5-dB increments.

### 4.2. Validation of Frame Synchronization

The frame synchronization process in the proposed method was evaluated by carrying out a frame synchronization test. The accuracy of frame synchronization $A_{cc}$ was considered to be correctly frame synchronized at 100%. This is defined as

(15) $$\begin{array}{c} A_{cc}=\frac{I_{\mathrm{CA}}}{I_{\mathrm{ALL}}}\times 100, \end{array}$$

where $I_{\mathrm{CA}}$ is the number of analysis frames that can be completely frame-synchronized, and $I_{\mathrm{ALL}}$ is the number of all analysis frames. The number of reference frames is 1, 2, 4, 8, 16, 32, 64, 128, and All (202). The total number of speech signal frames used in this evaluation is K/2, that is, 202.

**Figure 5:**
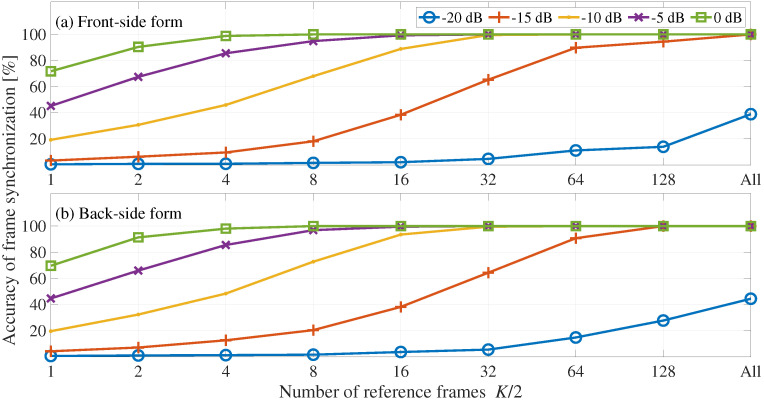
Accuracy of frame synchronization with respect to the number of reference frames: (**a**) front-side and (**b**) back-side forms. Each line shows the results for a different embedding-strength level $L_{\mathrm{SSL}}$.

Figure 5 shows the averaged accuracy of frame synchronization, where the horizontal axis represents the number of references frames and the vertical axis represents the accuracy of frame synchronization. Figure 5 shows the front-side and back-side forms. We can see here that the frame synchronization processes of the front-side and back-side forms have no significant difference in the accuracy of frame synchronization. In addition, the accuracy of frame synchronization increases with the increase of the number of reference frames and embedding-strength level. Specifically, the frame synchronization processes of the front-side and back-side forms have 100% accuracy of frame synchronization when the embedding-strength level is −15 dB and the number of reference frames is K/2, that is, 202.

Next, we evaluated the frame synchronization process of the front-side and back-side forms for robustness to the sample-cut attack. The first few samples of the signal with embedded bit strings are randomly deleted in the range of 1∼2N. The signal is utilized as input to analyze the accuracy of frame synchronization using Equation (15). The number of samples to be deleted is 14, 57, 109, 127, 144, 320, 406, 439, 480, 487, 494, and 611.

**Figure 6:**
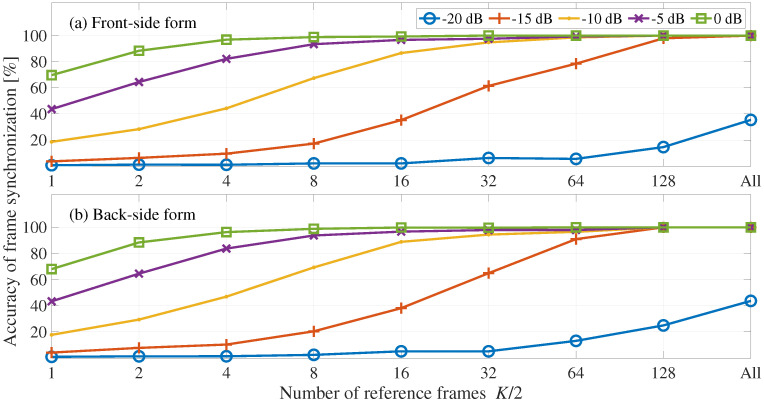
Evaluation of frame synchronization for desynchronization attacks: (**a**) front-side and (**b**) back-side forms. Each line shows the results for a different embedding-strength level $L_{\mathrm{SSL}}$.

Figure 6 shows the averaged accuracy of frame synchronization, where the horizontal axis represents the number of references frames K/2 and the vertical axis represents the accuracy of frame synchronization. Figure 6 shows the front-side and back-side forms. We can see here that the frame synchronization process of the front-side and back-side forms have no significant difference in the accuracy of frame synchronization. In addition, the accuracy of frame synchronization increases with the increase of the number of reference frames and embedding-strength level. Specifically, the frame synchronization accuracy in the front-side and back-side forms is 100% when the number of reference frames is All and the embedding-strength level is −15 dB.

### 4.3. Validation of Blind Detection

The performance of the blind detection process to detect security codes correctly was evaluated from the results of bit detection tests. The criterion for detecting a correct security code was a BER of less than or equal to 10%.

**Figure 7:**
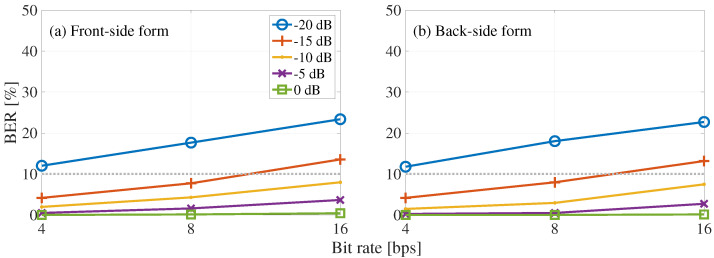
BER of proposed blind detection with respect to bit rate: (**a**) front-side and (**b**) back-side forms. Each line shows the results for a different embedding-strength level $L_{\mathrm{SSL}}$.

Figure 7 shows the BER results of blind detection concerning the bit rate, where the horizontal axis represents bit rate and the vertical axis represents BER. Figure 7 shows the front-side and back-side forms. The solid and dotted lines shows results with the BER of less than and greater than 10%, respectively. As we can see, the BER increases with the bit rate increase. In addition, a decrease in BER was observed with an increase in the embedding-strength level. As a result, we confirmed that at bit rates of 4 and 8 bps, the BER was less than 10% at the embedding-strength level of −15 dB, and at the bit rate of 16 bps, the BER was less than 10% at the embedding-strength level of −10 dB.

### 4.4. Validation of Blind Detection with Frame Synchronization

To investigate the effect of the frame synchronization process on the blind-detection process, we compared the BER of the blind-detection process alone to that with the frame synchronization. Here, we used the number of reference frames All, where the frame synchronization accuracy was 100%.

**Figure 8:**
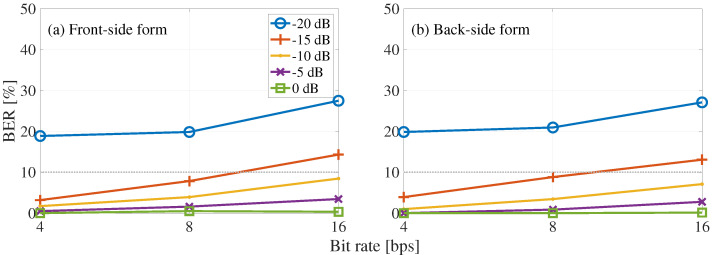
BER of proposed blind detection with frame synchronization regarding bit rate: (**a**) front-side and (**b**) back-side forms. Each line shows the results for a different embedding-strength level $L_{\mathrm{SSL}}$.

Figure 8 shows the BER results of the blind-detection process with the frame synchronization, where the horizontal axis represents bit rate and the vertical axis represents BER. Figure 8 shows the front-side and back-side forms. Comparing Figure 7 and Figure 8, we can see that the blind-detection process with frame synchronization reduced the BER by 9% at the embedding-strength level of −20 dB. On the other hand, at −15 to 0 dB, where the accuracy of the frame synchronization was 100%, no significant difference was observed with the presence or absence of frame synchronization.

### 4.5. Setting of Embedding Strength

The embedding strength level $L_{\mathrm{all}}$ in the proposed method is determined to minimize the PESQ, LSD, and BER simultaneously. As shown in Equation (6), the embedding strength is calculated from the power level of the host signal, the watermarked signal, and the embedding-strength level $L_{\mathrm{SSL}}$.

Bit detection and sound quality tests were carried out to determine the optimal $L_{\mathrm{SSL}}$. In the sound quality test, the PESQ International Telecommunication Union Telecommunication Standardization Sector ( ITU-T) P.862 and LSD were used as objective measures, as in our previous study [15]. In speech watermarking, it is generally helpful if the security code can be detected with a bit rate of 6 bps and a BER of less than or equal to 10%.

We carried out the sound quality tests (PESQ and LSD) to determine how well the watermarked signal was perceived. PESQ is the perceived sound quality of the watermarked signal relative to the original signal, expressed as mean opinion scores (MOS). The MOS varies on a scale of 1 (bad), 2 (poor), 3 (fair), 4 (good), and 5 (excellent). Typically, the PESQ threshold for speech watermarking is 3 (fair or slightly annoying). LSD is defined as

(16) $$\begin{array}{c} \mathrm{LSD}\left(X,Y\right)=\sqrt{\frac{1}{Q}\sum_{q=1}^{Q}(10\log_{10}\frac{{\left|X\left(\omega ,q\right)\right|}^{2}}{{\left|Y\left(\omega ,q\right)\right|}^{2}}{)}^{2}}, \end{array}$$

where X(ω,q) and Y(ω,q) are short-term Fourier transforms of the original signal and watermark signal, x(t) and y(t), in the *q*-th frame. Typically, the LSD criterion for speech watermarking is less than or equal to 1 dB.

**Figure 9:**
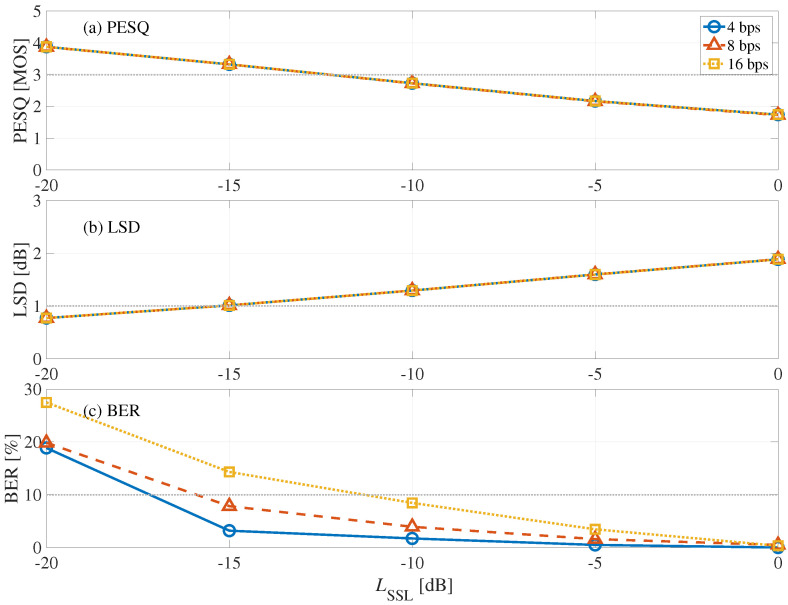
Relationship between embedding-strength level $L_{\mathrm{SSL}}$ of front-side form: (**a**) LSD, (**b**) PESQ, and (**c**) BER.

Figure 9 shows the results of PESQ, LSD, and BER from the blind detection process with frame synchronization of the front-side form, where the horizontal axis represents embedding-strength level $L_{\mathrm{SSL}}$ and the vertical axis represents (a) PESQ, (b) LSD, and (c) BER. As we can see, the BER decreases as $L_{\mathrm{SSL}}$ increases, and distortions increase as $L_{\mathrm{SSL}}$ increases. The $L_{\mathrm{SSL}}$ was determined to be −15 dB when the bit rate was 4 and 8 bps. At this time, the mean $L_{\mathrm{all}}$ was −43.7 dB. When the bit rate was 16 bps, there was no optimal $L_{\mathrm{all}}$. Thus, the $L_{\mathrm{SSL}}$ was determined to be −10 dB, in which BER <10%, LSD <2 dB, and PESQ 2 MOSs, at this bit rate. Hence, the mean $L_{\mathrm{all}}$ was −38.7 dB.

**Figure 10:**
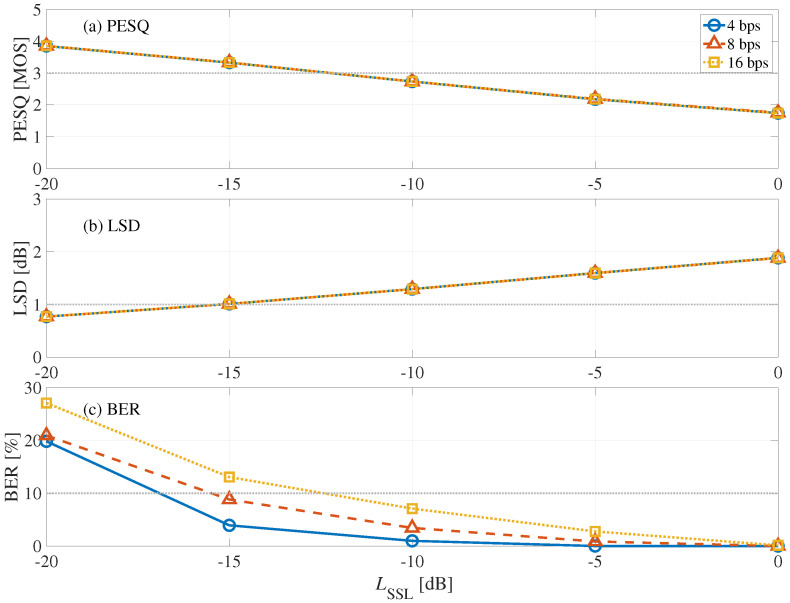
Relationship between embedding-strength level $L_{\mathrm{SSL}}$ of back-side form: (**a**) LSD, (**b**) PESQ, and (**c**) BER.

Figure 10 shows the results of PESQ, LSD, and BER from the blind detection process with frame synchronization of the back-side form, where the horizontal axis represents embedding-strength level $L_{\mathrm{SSL}}$ and the vertical axis represents (a) PESQ, (b) LSD, and (c) BER. We can see here that the BER decreases as $L_{\mathrm{SSL}}$ increases, and distortions increase as $L_{\mathrm{SSL}}$ increases. The $L_{\mathrm{SSL}}$ was determined to be −15 dB when the bit rate was 4 and 8 bps. At this time, the mean $L_{\mathrm{all}}$ was −43.7 dB. When the bit rate was 16 bps, there was no optimal $L_{\mathrm{all}}$. Thus, the $L_{\mathrm{SSL}}$ was determined to be −10 dB, in which BER <10%, LSD <2 dB, and PESQ 2 MOSs, at this bit rate. Hence, the mean $L_{\mathrm{all}}$ was −38.7 dB.

After applying the two embedding processes for the blind detection process with the frame synchronization process, the optimal embedding-strength level $L_{\mathrm{SSL}}$ was determined to be −15 dB.

## 5. Comparative Evaluations

We investigated whether the proposed method satisfies the four requirements of inaudibility, robustness, blind-detectability, and confidentiality by comparing it with the LSB, DSS [4], and MC [14] methods.

### 5.1. Database and Conditions

We selected a total of 100 utterances: 50 semi-randomly selected utterances from LibriSpeech [17] and 50 semi-randomly selected utterances from Voice Cloning Toolkit (VCTK) [18]. The selections were semi-random so that we could obtain utterances from a specific number of speakers. LibriSpeech was originally sampled at 16 kHz to study automatic speech recognition, and VCTK was originally sampled at 48 kHz to study speech synthesis. The sampling frequency for both databases was standardized to 16 kHz.

Inaudibility was evaluated by performing a sound quality test. Robustness was evaluated by carrying out basic robustness tests of ten processes as non-malicious interference [19]: normal (no interference), downsampling to 12 kHz (Resample-12), upsampling to 24 kHz (Resample-24), bit compression to 8 bit (Requant-8), bit expansion to 24 bit (Requant-24), conversion to Ogg format (Ogg), conversion to G711 coding (G711), conversion to G723.1 coding (G723.1), conversion to G726 coding (G726), and conversion to MP4 format (MP4). Blind-detectability was evaluated by carrying out frame synchronization tests and bit detection tests. Confidentiality was evaluated by performing bit detection tests when the embedding process and detection process of the front-side form and back-side form were switched. The bit rates of 4, 8, and 16 were used for evaluation.

### 5.2. Evaluation Results for Inaudibility

**Figure 11:**
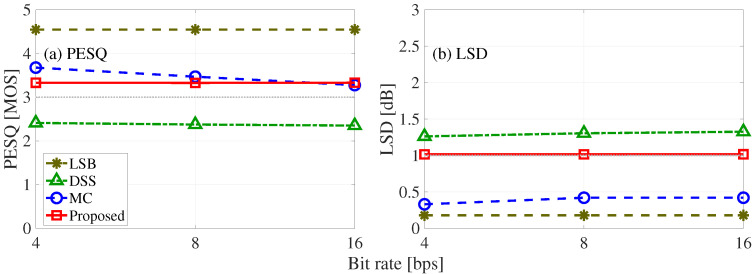
Inaudibility results of four compared methods (Proposed, MC, DSS, and LSB): (**a**) LSD and (**b**) PESQ.

Figure 11 shows the evaluation results, namely, PESQ and LSD with respect to the bit rate, for the sound quality test of the LSB, DSS, MC, and proposed methods. As we can see, the LSB method has the best sound quality among the four methods. The DSS method has the PESQ of less than 3 and the LSD of more than 1 dB. The PESQ for the MC method is greater than 3 to 16 bits, and the LSD is less than 1 dB. The PESQ for the proposed method is greater than 3 until 8 bits, and the LSD is 1 dB.

### 5.3. Evaluation Results for Robustness

**Figure 12:**
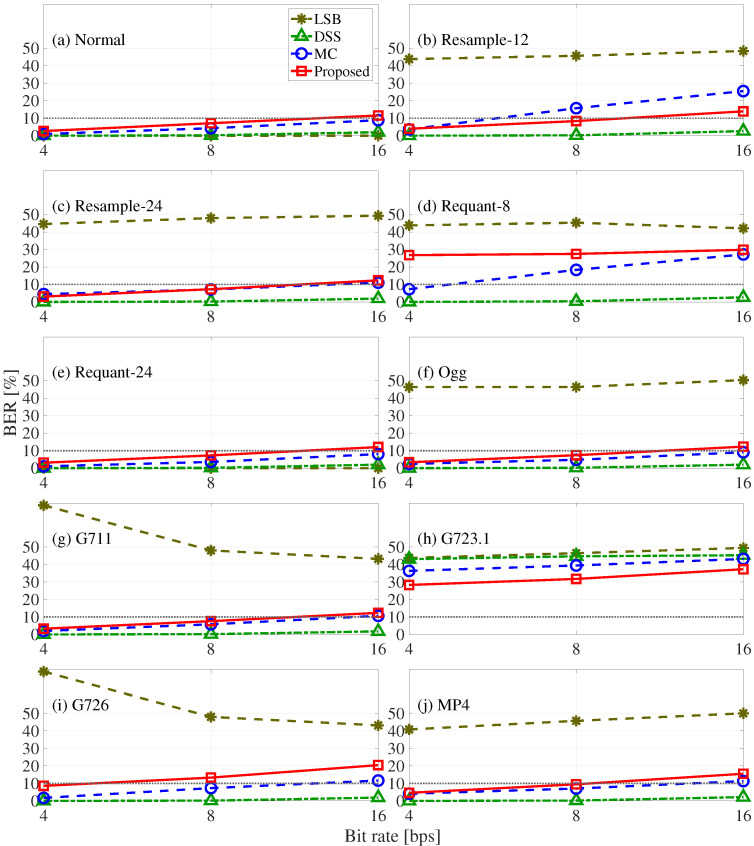
Robustness results of four compared methods (Proposed, MC, DSS, and LSB) in terms of BER in ten cases: (**a**) normal, (**b**) resample-12, (**c**) resample-24, (**d**) requant-8, (**e**) requant-24, (**f**) Ogg, (**g**) G711, (**h**) G723.1, (**i**) G726, and (**j**) MP4.

Figure 12 shows the evaluation results for the basic robustness tests of the LSB, DSS, MC, and proposed methods, where the horizontal axis shows the bit rate and the vertical axis shows the BER of each attack process. As we can see, the LSB method is not robust (i.e., does not have a BER greater than 10%) against any attacks except Requant-24. The DSS method is robust (BER less than 10%) except for G723.1. The MC method and the proposed method are robust (BER less than 10%) except for the Requant-8 and G723.1 encodings. Unfortunately, speech watermarking using LP residuals is fundamentally vulnerable to G726.

### 5.4. Evaluation Results for Blind-Detectability

Blind-detectability was evaluated by carrying out the frame synchronization test and bit detection test of the blind detection process with the frame synchronization process when the embedding-strength level was varied. In this paper, we regard blind-detectability to be achieved if the following two conditions are satisfied: (a) the BER is less than or equal to 10% and (b) the accuracy of the frame synchronization is greater than or equal to 80%. The magenta marker shows the result when the embedding-strength level $L_{\mathrm{SSL}}$ is −15 dB.

**Figure 13:**
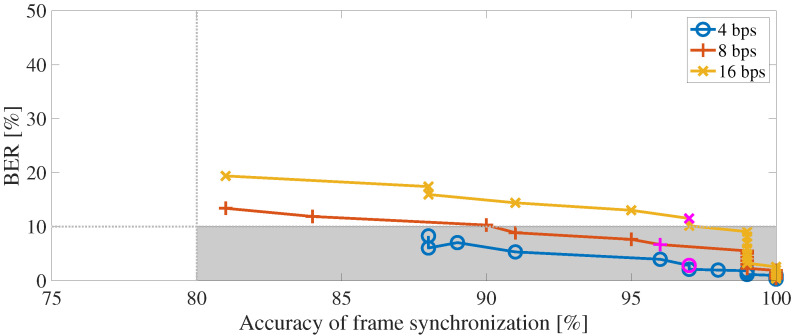
BER with respect to the accuracy of frame synchronization. Gray shading indicates areas that meet criterion of BER of less than 10% and frame synchronization accuracy greater than 80%.

Figure 13 shows the results of blind-detectability, where the horizontal axis represents the accuracy of frame synchronization and the vertical axis represents BER. The gray areas indicate that two conditions can be satisfied simultaneously. We can see here that the BER decreases as the accuracy of the frame synchronization increases. The results at 4 and 8 bps show that the accuracy of the frame synchronization is greater than 80%, and the BER of less than 10% is satisfied at the embedding-strength level $L_{\mathrm{SSL}}$ of −15 dB.

### 5.5. Evaluation Results for Confidentiality

The proposed method can increase confidentiality in non-public conditions. However, if it were to be implemented in the public condition, we do not know what would happen to the confidentiality. Therefore, confidentiality was evaluated by carrying out bit detection tests when the embedding and detection processes were switched in public conditions. Confidentiality was defined as the security codes being incorrectly detected if the BER was greater than or equal to 20%.

**Figure 14:**
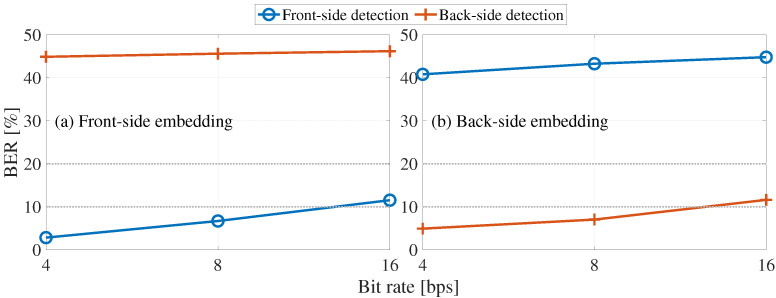
Results of BER by swapping embedding and detection processes: (**a**) front-side and (**b**) back-side forms.

Figure 14 shows the evaluation results of the confidentiality, where the horizontal axis shows the bit rate and the vertical axis shows the BER of each attack process. We can see here that embedding by the front-side form does not allow the back-side form to detect the security codes correctly. Moreover, when embedded by the back-side form, the security codes could not be detected correctly by the front-side form. If a security code is embedded in the front-side form, the back-side form cannot properly frame-synchronize because the amount of remaining LP used in the front-side and back-side forms is different. Therefore, if embedding and detection are performed on different forms, frame synchronization will not be possible and the BER of security codes will be increased. These results demonstrate that the proposed method is confidential in public conditions thanks to the front-side and back-side forms.

### 5.6. Performance Limitations

The proposed method has two limitations: (i) the accuracy of frame synchronization due to the number of reference frames depending on the signal length and (ii) limited embedding capacity due to the frame length of the LP analysis.

For (i), the evaluation in Section 4.2 shows that the accuracy of frame synchronization depends on the number of reference frames. At least 128 reference frames are required for frame synchronization accuracy to exceed 80%, which means the frame window length of 20 ms would require at least 5.12 s for the host signal.

For (ii), we used a frame window length of 20 ms for the proposed method as the commonly used analysis length for LP analysis. In that case, the number of frames per second is 50. Since the proposed method embeds one bit in two frames, the embedding capacity is limited to 25 bps. Generally, the bit rate of 4 bps is sufficient for speech watermarking. When speech watermarking is applied to tampering detection and spoofing detection, the precision of the tampering detection and spoofing detection depends on the bit rate. If the precision of 0.125 is required, a bit rate of at least 16 bps is needed.

These limitations are due to the fact that the frame length is 20 ms. Reducing the frame length to shorter than 20 ms should lead to an increase in the number of frames, which in turn will lead to an increase in the embedding capacity and the number of reference frames.

## 6. Conclusions

We developed a state-of-the-art speech watermarking technique called the LP-DSS scheme that satisfies the four requirements of inaudibility, robustness, blind-detectability, and confidentiality. For blind-detectability and confidentiality, we proposed two forms of data embedding for blind detection with frame synchronization, and we investigated ways of mitigating the trade-off between inaudibility and watermark detection rate by varying the embedding-strength level. The evaluation results showed that, with the embedding-strength level of −15 dB, the proposed method satisfies the inaudibility and watermark detection rate requirements until 8 bps. We also found that the proposed method is inaudible and robust in detecting security codes, and that it has sufficient capability for blind detection. Regarding confidentiality, we demonstrated that if the security code is embedded on the front-side or back-side form, and vice versa, it cannot be detected.

## Data Availability

Not applicable.

## References

[1] Todisco M., Wang X., Vestman V., Sahidullah M., Delgado H., Nautsch A., Yamagishi J., Evans N.W.D., Kinnunen T.H., Lee K.A. ASVspoof 2019: Future Horizons in Spoofed and Fake Audio Detection. Proceedings of the 20th Annual Conference of the International Speech Communication Association (INTERSPEECH 2019).

[2] Wu Z. (2014). Information Hiding in Speech Signals for Secure Communication.

[3] Cvejic N., Seppanen T.L. (2007). Digital Audio Watermarking Techniques and Technologies.

[4] Lin Y., Abdulla W. (2015). Audio Watermark: A Comprehensive Foundation Using MATLAB.

[5] Unoki M., Miyauchi R. (2015). Robust, Blindly-detectable, and Semi-reversible Technique of Audio Watermarking Based on Cochlear Delay. IEICE Trans. Inf. Syst..

[6] Hua G., Huang J., Shi Y.Q., Goh J., Thing V.L.L. (2016). Twenty Years of Digital Audio Watermarking—A Comprehensive Review. Signal Process..

[7] Boney L., Tewfik A.H., Hamdy K.N. Digital Watermarks for Audio Signals. Proceedings of the Third IEEE International Conference on Multimedia Computing and Systems.

[8] Bhat V.K., Sengupta I., Das A. (2011). An Audio Watermarking Scheme Using Singular Value Decomposition and Dither Modulation Quantization. Multimed. Tools Appl..

[9] Lei B., Soon I.Y., Tan E.L. (2013). Robust SVD-Based Audio Watermarking Scheme with Differential Evolution Optimization. IEEE Trans. Audio Speech Lang. Process..

[10] Ngo M.N., Unoki M. (2016). Method of Audio Watermarking Based on Adaptive Phase Modulation. IEICE Trans. Inf. Syst..

[11] Wang S., Unoki M. Hybrid Speech Watermarking Based on Formant Enhancement and Cochlear Delay. Proceedings of the 2014 Tenth International Conference on Intelligent Information Hiding and Multimedia Signal Processing.

[12] Wang S., Yuan W., Wang J. (2019). Speech Watermarking Based on Source-flter Model of Speech Production. J. Inf. Hiding Multimed. Signal Process..

[13] Wang S., Unoki M. (2015). Speech Watermarking Method Based on Formant Tuning. IEICE Trans. Inf. Syst..

[14] Mawalim C.O., Unoki M. (2021). Speech Watermarking Method Using McAdams Coefficient Based on Random Forest Learning. Entropy.

[15] Namikawa R., Unoki M. (2020). Non-Blind Speech Watermarking Method Based on Spread-Spectrum Using Linear Prediction Residue. IEICE Trans. Inf. Syst..

[16] Takeda K., Sagisaka Y., Katagiri S., Abe M., Kuwabara H. (2010). Speech Database User’s Manual.

[17] Panayotov V., Chen G., Povey D., Khudanpur S. Librispeech: An ASR corpus based on public domain audio books. Proceedings of the 2015 IEEE International Conference on Acoustics, Speech and Signal Processing (ICASSP 2015).

[18] Veaux C., Yamagishi J., Macdonald K. (2017). CSTR VCTK Corpus: English Multi-Speaker Corpus for CSTR Voice Cloning Toolkit. https://datashare.ed.ac.uk/handle/10283/3443.

[19] Information Hiding and Its Criteria for Evaluation. https://www.ieice.org/iss/emm/ihc/.

